# Fluorinated Fullerenes as Electrolyte Additives for High Ionic Conductivity Lithium-Ion Batteries

**DOI:** 10.3390/molecules29132955

**Published:** 2024-06-21

**Authors:** Haoyu Pan, Zhanlin Yang, Jianhui Chen, Hengyi Li, Cuilian Wen, Baisheng Sa

**Affiliations:** 1 Multiscale Computational Materials Facility & Materials Genome Institute, School of Materials Science and Engineering, Fuzhou University, Fuzhou 350108, China; 182100321@fzu.edu.cn (H.P.); 211820067@fzu.edu.cn (Z.Y.); n231810008@fzu.edu.cn (J.C.); clwen@fzu.edu.cn (C.W.); 2 Fujian Applied Technology Engineering Center of Power Battery Materials, Fujian College of Water Conservancy and Electric Power, Yong’an 366000, China

**Keywords:** additives, density functional theory, fullerenes, first-principle calculations, molecular dynamics, electrolytes

## Abstract

Currently, lithium-ion batteries have an increasingly urgent need for high-performance electrolytes, and additives are highly valued for their convenience and cost-effectiveness features. In this work, the feasibilities of fullerenes and fluorinated fullerenes as typical bis(fluorosulfonyl)imide/1,2-dimethoxymethane (LiFSI/DME) electrolyte additives are rationally evaluated based on density functional theory calculations and molecular dynamic simulations. Interestingly, electronic structures of C_60_, C_60_F_2_, C_60_F_4_, C_60_F_6_, 1-C_60_F_8_, and 2-C_60_F_8_ are found to be compatible with the properties required as additives. It is noted that that different numbers and positions of F atoms lead to changes in the deformation and electronic properties of fullerenes. The F atoms not only show strong covalent interactions with C cages, but also affect the C-C covalent interaction in C cages. In addition, molecular dynamic simulations unravel that the addition of trace amounts of C_60_F_4_, C_60_F_6_, and 2-C_60_F_8_ can effectively enhance the Li^+^ mobility in LiFSI/DME electrolytes. The results expand the range of applications for fullerenes and their derivatives and shed light on the research into novel additives for high-performance electrolytes.

## 1. Introduction

In the context of the growing prevalence of electrification and the development of renewable energy sources, electrochemical energy storage technology is becoming an increasingly pivotal solution to the challenges associated with energy storage and conversion [1,2,3]. One of the most crucial performance parameters in electrochemical energy storage devices is the optimization and enhancement of ionic conductivity [4], which has emerged as a significant area of research and development [5,6,7]. Ionic conductivity not only directly affects the charging and discharging rate, energy density, and cycle life of devices such as batteries and supercapacitors, but also determines the operating stability of the devices at different temperatures [8,9]. Therefore, research on the enhancement of ionic conductivity is imperative to gain a deeper understanding and to optimize electrochemical energy storage technologies, thereby providing more effective solutions for sustainable energy transitions.

Additives play a vital role in electrolyte systems and are widely used in a variety of electrochemical devices and systems, including lithium-ion batteries, fuel cells, and supercapacitors [10,11,12,13]. The incorporation of additives can remarkably enhance the functionality of electrolytes, conferring a multitude of benefits and capabilities. Firstly, the addition of appropriately selected additives can enhance the ion transport properties of the electrolyte, thereby increasing the mobility of ions through the electrolyte. This, in turn, can improve the efficiency and kinetic response of the electrochemical reaction [14]. It thereby facilitates the enhancement of the energy density, power density, and cycle stability of the battery or electrochemical system [15,16]. In addition, some additives can have a significant effect on the electrode, forming a protective layer on the electrode surface to reduce electrolyte decomposition and electrode corrosion, thus further optimizing the electrochemical performance [17]. Moreover, additives are employed to enhance the high-temperature, freezing, or flame-retardant properties of electrolytes, enabling them to meet specific application requirements. One such additive is capable of releasing free radicals that interrupt the combustion chain reaction by trapping hydrogen or hydroxyl radicals in the gas phase, thus achieving flame-retardant properties [18,19,20]. However, the search for ideal additives that play an irreplaceable role in electrolyte systems by providing key support for the performance enhancement and optimization of electrochemical devices, improving ionic transport properties, modulating physicochemical properties, and providing specific functionality and protection mechanisms is still ongoing [21]. It is important that future research further explores the design and application of novel additives to meet the needs of the evolving field of electrochemistry.

The fullerene family and its derivatives exhibit distinctive physicochemical properties and three-dimensional topologies, rendering them highly effective in a diverse array of applications [22,23,24]. Due to its significant polarizability and very high electronegativity, the introduction of a F atom into organic molecules can lead to a significant electron distribution shift, which in turn affects the neighboring groups as well [25,26]. In addition, the F element has relatively high electron affinity energy compared with other elements, and the access to fluorine atoms on fullerene molecules can result in the formation of significant C-F bonds, which can directly change the electronic properties of fullerenes [25]. Therefore, fluorinated fullerenes exhibit peculiar structures and unique properties, which have attracted the interest of a wide range of researchers and scholars [27,28]. In recent years, there has been a great deal of interest in the research area of improving the performance of LIBs by introducing fluoride or generating fluoride intermediates. For instance, it is proposed to stabilize lithium metal batteries by means of the local redistribution of fluoride, which enables the active lithium metal to be locked in place [29]. Pentafluorophenylboron oxalate (PFPBO) has been synthesized and used as an additive in electrolytes to exhibit high ionic conductivity [30]. However, the understanding of fluorinated fullerenes as additives for electrolytes is not complete. In addition, accelerating materials development and exploring mechanisms by means of computational simulation is widely used in many studies [31,32,33]. Therefore, a systematic study of fluorinated fullerenes as additives based on advanced computational simulation is of great interest to promote the development and research of high-performance electrolytes and to provide theoretical guidance.

This study presents for the first time the introduction of small amounts of fullerene and fluorinated fullerene as additives in bis(fluorosulfonyl)imide/1,2-dimethoxymethane (LiFSI/DME) electrolytes to design the electrolyte and to comprehensively analyze the improvement of its performance using density functional theory (DFT) calculations and molecular dynamics (MD) simulations. The effects of fullerene and fluorinated fullerene on LiFSI/DME electrolytes are fully elucidated. The study provides accurate electronic structure information and description of the dynamic behavior. It is worth highlighting that although the electronic properties exhibited by the selected fullerenes and several fluorinated fullerenes in the study meet the requirements to be used as additives to LiFSI/DME electrolytes, after actual addition to the LiFSI/DME electrolyte, the MD results show that only some of the fluorinated fullerenes have an enhancement effect on the lithium ions’ mobility. This study demonstrates the potential of fluorinated fullerenes as electrolyte additives, a discovery with significant implications for the advancement of electrochemical energy storage technology. It not only provides theoretical guidance and technical support for the development and improvement of new electrolytes, but also broadens the application fields of fullerenes and their derivatives.

## 2. Results and Discussion

**Figure 1:**
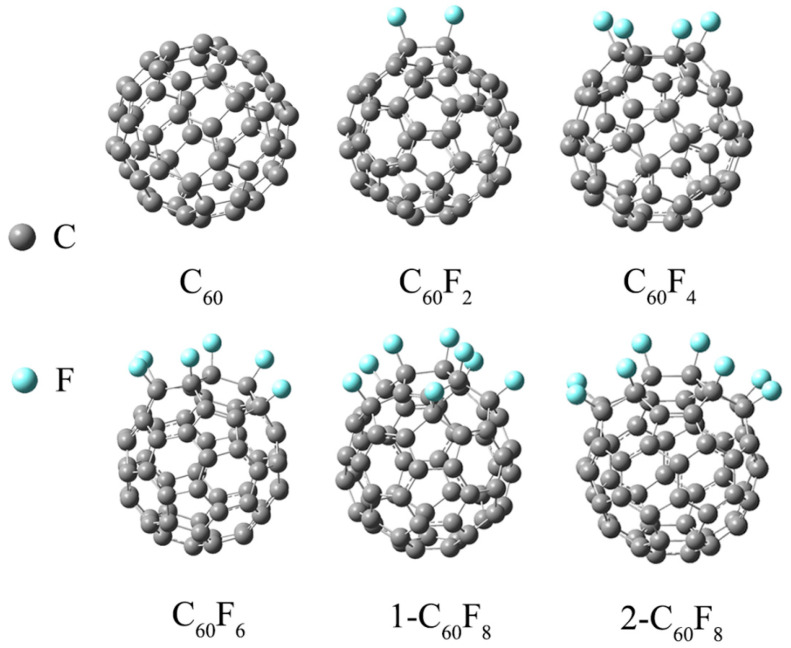
Optimized geometry of fullerene C_60_ and fluorinated fullerene.

Figure 1 shows the most stable structures for C_60,_ C_60_F_2_, C_60_F_4_, C_60_F_6_, 1-C_60_F_8_, and 2-C_60_F_8_ after full optimization. The fullerenes selected for our study were all successfully isolated, and the bonding sites of the F atoms were identified [34]. It can be seen from Figure 1 that the introduction of different numbers of F atoms and different sites leads to different degrees of changes in the shape of the initial fullerene carbon cage. For example, although 1-C_60_F_8_ and 2-C_60_F_8_ have the same number of fluorine atoms, the different positions of the F atoms on the C cage result in different deformations of the fluorinated fullerene, potentially giving them different properties.

**Figure 2:**
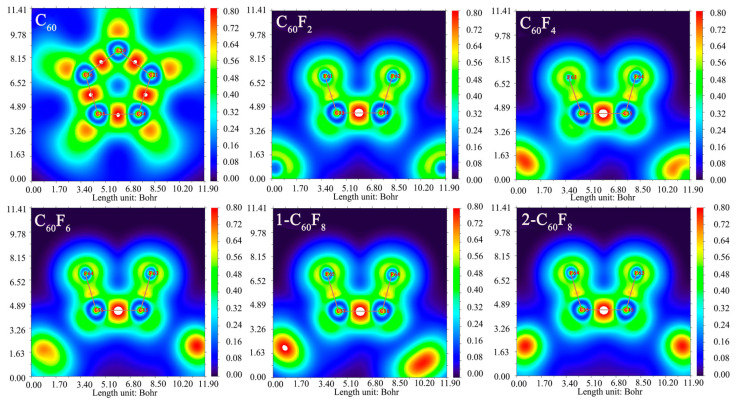
Two-dimensional localized orbital locator (LOL) map of fullerenes and fluorinated fullerenes. The slice plane is defined by the C and F atoms.

In order to probe the effect of the introduction of F atoms in depth, a localized orbital locator (LOL) map analysis was performed (shown in Figure 2) for six structures using DFT simulations to analyze the interaction of the introduced F atoms with the carbon cages as well as the effect of this interaction on the C-C covalent bonding between the initial carbon cages. Based on the results in Figure 2, the LOL data between the C-C atoms shows highly localized regions, which suggests strong covalent interactions between the C-C atoms. Whereas the highly localized regions between the F atoms and C atoms appear to be relatively weak compared with those between the C-C atoms, there are still strong localized regions, which suggests covalent interactions between C-F atoms as well. Interestingly, both before and after the introduction of F atoms, the C-C in the fullerene cage shows highly localized regions that retain covalent interactions. These results provide important clues for further understanding the effects of introducing F atoms on molecular structures and interactions, which is helpful to reveal the properties and potential applications of these compounds.

**Figure 3:**
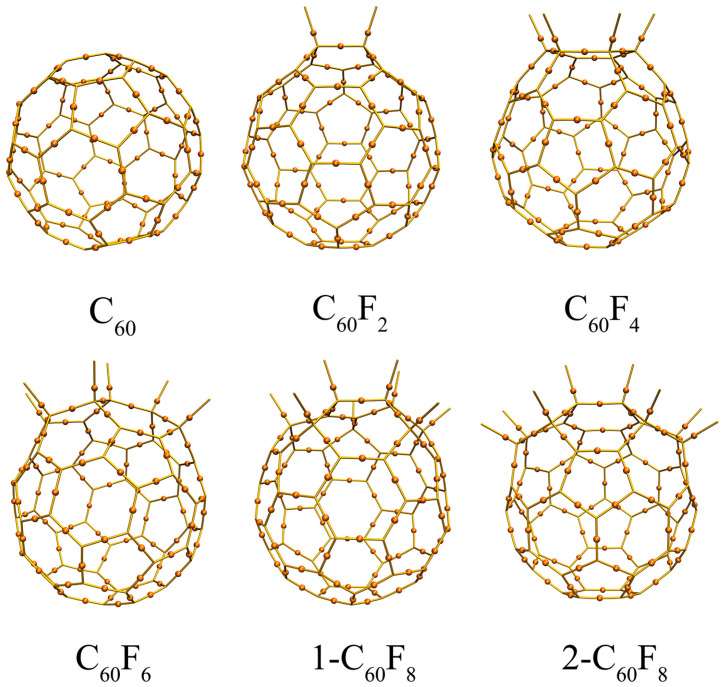
Critical points of (3, −1) for fullerene and fluorinated fullerenes.

In order to deeply explore the strong covalent interactions between F and C atoms as well as between C and C atoms in C_60_, C_60_F_2_, C_60_F_4,_ C_60_F_6_, 1-C_60_F_8_, and 2-C_60_F_8_, bond critical point (BCP) and Mayer bond order (MBO) calculations were also performed. Figure 3 illustrates the distribution of critical points, while Table 1 lists the bond descriptors for the BCPs and MBO results. For all six cases, the calculations observe *ρ*_BCP_ > 0, *H*_BCP_ < 0, |*V*_BCP_|/*G*_BCP_ > 2, and MBO > 0.8, and these results indicate the presence of strong covalent interactions between carbon atoms [35]. Similarly, significant covalent interactions exist between C and F atoms. It is noteworthy that F atoms lead to noticed changes in the values of ρ_BCP_, |*V*_BCP_|/*G*_BCP_, and MBOs between C-C, further demonstrating that the introduction of F atoms changes the strength of covalent interactions between C-C to some extent.

**Figure 4:**
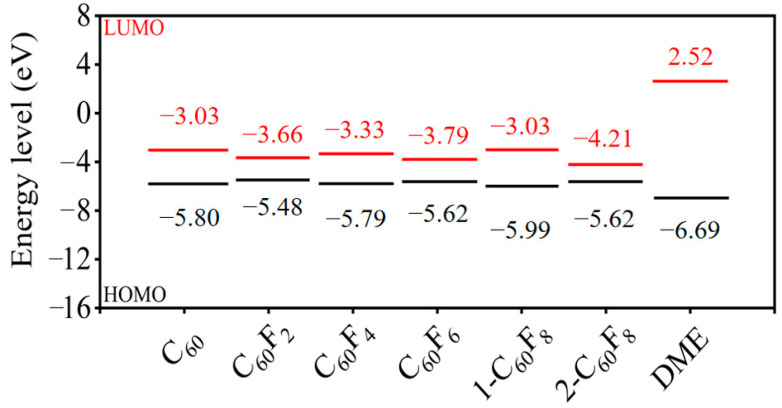
HOMO and LUMO energy levels for fullerene, fluorinated fullerene, and solvent DME.

To analyze whether fullerenes and fluorinated fullerenes have the potential to be applied as additives to DME-based electrolytes in terms of their electronic energy levels, we have calculated the molecular orbitals of fullerenes, fluorinated fullerenes, and the electrolyte solvent DME. Figure 4 represents the highest occupied molecular orbital (HOMO) and the lowest unoccupied molecular orbital (LUMO) for each fullerene and fluorinated fullerene. The HOMO–LUMO energy levels of solvent DME were also calculated as a comparison. From Figure 4, it can be seen that the fluorinated fullerenes with different degrees of fluorination show different electronic properties and are very obvious, reflecting from the side that the influence of F atoms on the initial fullerene carbon cage effect cannot be neglected. Even with the same number of F atoms introduced into the carbon cage, the different F atom sites result in different electronic energy levels. For example, the two fluorinated fullerenes 1-C_60_F_8_ and 2-C_60_F_8_ show LUMO levels of −3.00 and −4.21 eV, respectively.

Next, we theoretically assessed the feasibility of fullerenes and fluorinated fullerenes as additives to DME-based electrolytes by comparing their HOMO–LUMO energy levels with those of the solvent DME. The HOMO and LUMO energy levels of DME are −6.69 and 2.52 eV, respectively. In comparison, the HOMO energy levels of C_60_, C_60_F_2_, C_60_F_4_, C_60_F_6_, 1-C_60_F_8_, and 2-C_60_F_8_ are higher than the HOMO energy level of DME. This property causes them to be oxidized preferentially to the electrolyte solvent DME when used as an additive and form a protective layer on the cathode surface, which further improves its stability, resulting in the construction of lower-resistance cathode electrolyte interface (CEI) films. In addition, calculations showed that the LUMO energy levels of these compounds were lower than those of the solvent DME, suggesting that they were preferentially reduced over the DME molecules, thus facilitating the formation of insoluble solid electrolyte interphase (SEI) films on the anode surface. This effectively prevents solvent decomposition and greatly enhances the electrochemical performance of Li-ion batteries. From the global perspective of electronic energy levels, C_60_ and fluorinated fullerenes demonstrate the possibility of being used as additives in DME-based solvent electrolytes and may provide important reference ideas for battery performance enhancement.

**Figure 5:**
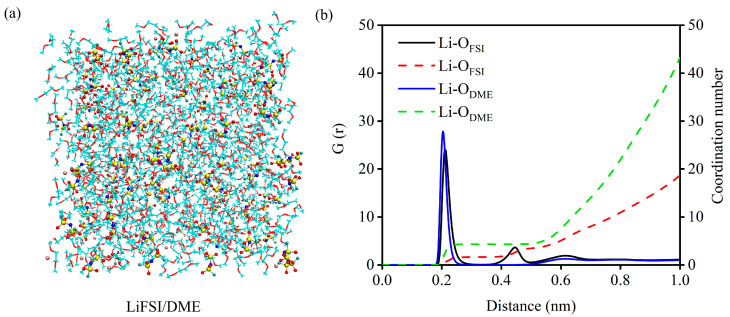
(**a**) Structure snapshot, (**b**) radial distribution function and coordination number of LiFSI/DME electrolyte model. The DME solvent molecules are shown in line models, and LiFSI molecules are shown in ball-and-stick models, the red balls are the oxygen atoms, the yellow balls are the sulfur atoms, the blue balls are the nitrogen atoms, the cyan balls are the fluorine atoms and the pink balls are the lithium atoms.

However, whether C_60_, C_60_F_2_, C_60_F_4_, C_60_F_6_, 1-C_60_F_8_, and 2-C_60_F_8_ as additives can play a positive role in the electrolyte still needs to be further explored and investigated in the actual electrolyte system. Herein, LiFSI/DME is used as the research object to construct a real-environment electrolyte system with different additives. We first performed MD simulations of the LiFSI/DME electrolyte to observe its own physicochemical properties and solvation structure, as shown in Figure 5a. Figure 5b presents the results of the radial distribution function (RDF) and coordination number (CN) of the electrolyte model. Based on our simulation results, we can observe that the distribution of the solvent DME in the first solvation layer is higher than that of the anionic FSI^−^ in the LiFSI/DME system, which provides an important initial reference for the further evaluation of the modulation of the electrolyte properties by fullerenes and fluorinated fullerenes as additives.

**Figure 6:**
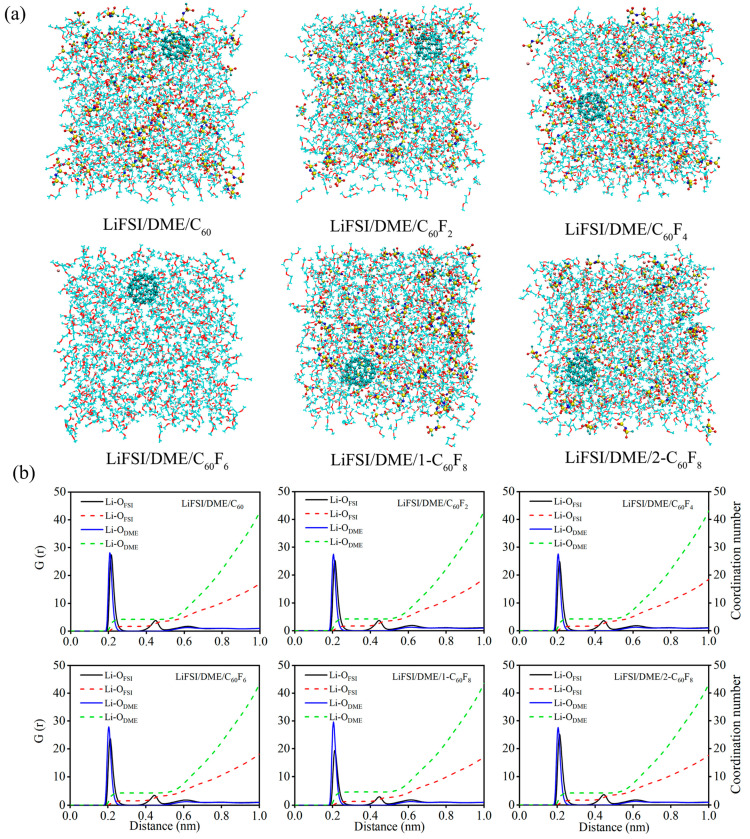
(**a**) Structure snapshots, (**b**) radial distribution functions and coordination numbers of Li+ with anions and solvents for different electrolytes. The DME solvent molecules are shown in line models, fullerene, fluorinated fullerenes and and LiFSI molecules are shown in ball-and-stick models, the red balls are the oxygen atoms, the yellow balls are the sulfur atoms, the blue balls are the nitrogen atoms, the cyan balls are the fluorine atoms and the pink balls are the lithium atoms.

Considering that C_60_, C_60_F_2_, C_60_F_4_, C_60_F_6_, 1-C_60_F_8_, and 2-C_60_F_8_ have the electronic properties to be used as additives in DME-based electrolytes, the additive performance was further carried out by doping these six substances one by one in LiFSI/DME electrolytes to form electrolyte systems for MD simulations. Figure 6 illustrates the constructed models and the RDF and CN of the six electrolytes after MD simulations. The simulation results demonstrate that fullerene and fluorinated fullerenes, when incorporated into the electrolytes as additives, do not significantly alter the RDF and CN of the LiFSI/DME electrolytes. The first solvation layer is still predominantly occupied by the solvent DME, whose concentration is significantly higher than that of the anionic FSI^−^. Nevertheless, the incorporation of diverse additives still exerted some subtle influences on the initial RDF and the C. In order to facilitate a more accurate comparison of the magnitude of the CN, the details of the electrolyte systems are presented in Table 2. Another interesting finding is that the CN of the solvent DME decreases when the coordination number of anionic FSI^−^ increases after the addition of additives, and vice versa; when the coordination number of anionic FSI^−^ decreases, the coordination number of solvent DME increases. Generally speaking, they show a kind of mutual constraint relationship.

**Figure 7:**
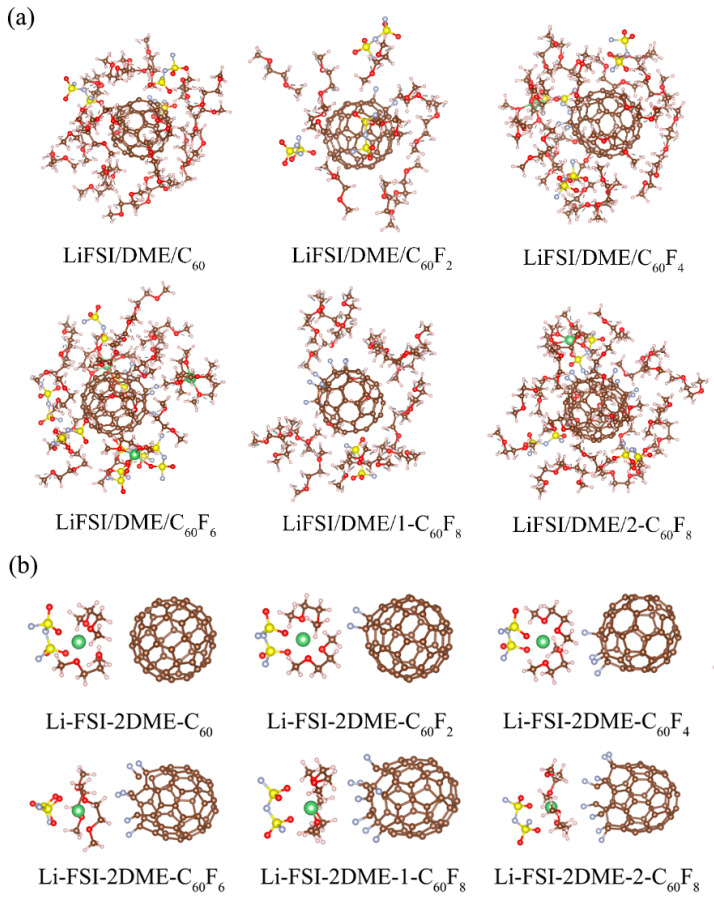
(**a**) Substances within 5 Å of the additive after MD equilibration. (**b**) Optimized solvation structures by DFT. The brown balls are carbon atoms, the gray balls are fluorine atoms, the red balls are oxygen atoms, the pink balls are hydrogen atoms, the yellow balls are sulfur atoms, and the green balls are the lithium atoms.

In order to show more clearly the environment of the solvation layer within a certain range around the additive, we extracted the MD snapshots of the electrolyte systems within a range of 5 Å around the additive in Figure 7. As can be seen, in the electrolyte systems, the additives are not directly involved in the solvation structure of the lithium ions, but they are surrounded by different solvation substances and some free molecules. This phenomenon suggests that the additives used in the study have electrolyte-compatible properties and are able to be incorporated into the electrolyte rather than being present in a separate free state. To further confirm the interaction between different fluorinated fullerenes and electrolytes, we performed DFT optimizations of the electrolyte systems for solvation environments around Li^+^ based on the ratio of anionic and solvent coordination numbers, as shown in Figure 7b. Herein, the accurate DFT data show that fullerenes and fluorinated fullerenes do not participate in the solvation layer of Li^+^, which is consistent with the results of the molecular dynamics simulations that we extracted.

**Figure 8:**
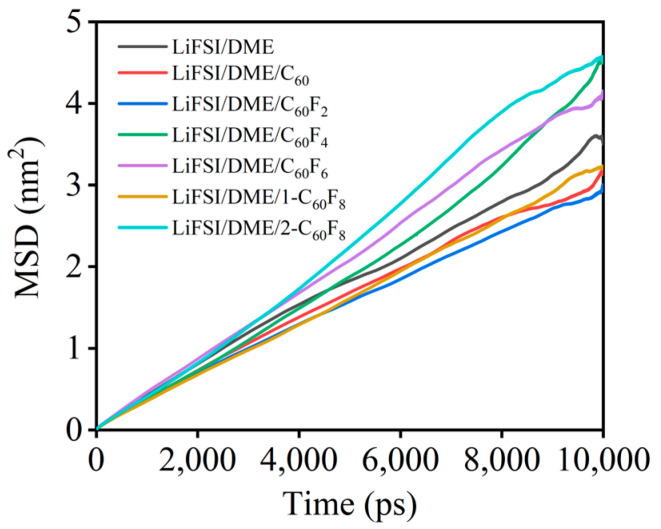
Mean square displacements of Li^+^ under different electrolytes.

Figure 8 shows the self-diffusion coeafficient of Li^+^ in different electrolytes obtained by MD simulations, a property that plays a crucial role in the ionic conductivity of LIBs. It is noted that the self-diffusion coefficients of Li^+^ are significantly different in the presence of different additives. The results of the MD simulations show that the migration properties of Li^+^ are significantly enhanced with the addition of partially fluorinated fullerene. Specifically, the diffusion coefficient of Li^+^ reached the maximum value in the LiFSI/DME/2-C_60_F_8_ electrolyte system, while different degrees of enhancement were also achieved in the LiFSI/DME/C_60_F_2_ and LiFSI/DME/C_60_F_4_ electrolyte systems, respectively. However, the effects of the other studied additives were less pronounced and even hindered Li^+^ migration to some extent. Overall, only C_60_F_4_, C_60_F_6_, and 2-C_60_F_8_ as the additives can significantly enhanced Li^+^ migration compared with the original LiFSI/DME electrolytes, suggesting their good application potential in practical electrolyte systems. Therefore, we found that the properties of fluorinated fullerenes can change significantly due to the number and position of the introduced fluorine atoms, and therefore, not all fluorinated fullerenes have a positive effect on the electrolyte system. The effects of specific substances need to be analyzed in detail in a real environment. Table 3 lists the electrolyte simulation systems and their properties as a summary, including details of the dimensions of the simulation box, the proportions of the components of the electrolyte, and the viscosity and diffusion coefficients.

## 3. Computational Details

The B3LYP exchange-correlation approximation and the 6-31g(d,p) basis set were utilized with hybrid DFT, and an implicit solvation model (Solvation Model Density, SMD, Carnegie Mellon University, Pittsburgh, PA, USA) was used [36]. The molecular structure of the electrolyte was optimized and analyzed by Gaussian16 [37] and Multiwfn 3.8 [38]. All calculations were performed using the GD3BJ correction. The dissolution structure of the electrolyte was derived from MD simulations using the GROMACS2018 (Berendsen Laboratory, University of Gottingen, Göttingen, Germany) software package [39], which is based on the OPLS-AA force field [40] generated with the help of AuToFF [41]. Modeling was performed with the help of PACKMOL v20.14.4 software (Herman Berendsen’s group, department of Biophysical Chemistry of Groningen University) [42]. The initial structure size used for the MD simulations was 10.0 × 10.0 × 10.0 nm. The particle-particle-grid method was included to treat the long-range Coulomb interactions. The van der Waals interactions were described using the Lennard-Jones interaction model. In order to eliminate as much as possible unreasonable configurations in the initial structure, a conjugate gradient energy minimization scheme was applied to the initial configurations. A 20 ns NPT simulation was first performed at 298.15 K to achieve full equilibrium. Then, 10 ns NVT simulations were performed at 298.15 K for data sampling. Finally, the electrolyte structure was visualized using VMD [43] and VESTA [44]. The mean square displacement (MSD) of Li^+^ was tracked during the simulations [45]. By analyzing the MSD results, the diffusion coefficient D of Li^+^ in the electrolyte were calculated according to the following equation:

(1) $$D=\frac{1}{6Na}\underset{t\to \infty }{\lim}\frac{d}{dt}\sum_{i=1}^{Na}{\left[ri(t)-ri(0)\right]}^{2}$$

where *N_α_* is the number of atoms, and *r_i_*(*t*) and *r_i_*(0) are the positions of atom *i* at times *t* and 0, respectively.

## 4. Conclusions

In summary, the structural and electronic properties of C_60_, C_60_F_2_, C_60_F_4_, C_60_F_6_, 1-C_60_F_8_, and 2-C_60_F_8_ are systematically investigated in this study using DFT calculations. The results show that the introduction of F atoms affects the initial structure of C_60_, leading to different degrees of deformation of the carbon cage, and there are certain covalent interactions between the introduced F atoms and the carbon cage. In addition, the introduction of F atoms into fullerenes will also have a certain effect on the strength of the initial C-C covalent interactions of fullerenes, which leads to significant differences in properties between different fluorinated fullerenes. By calculating the frontier molecular orbitals, we found that the HOMO–LUMO energy levels of C_60_, C_60_F_2_, C_60_F_4_, C_60_F_6_, 1-C_60_F_8_, and 2-C_60_F_8_ show potential for application as DME-based electrolyte additives. Furthermore, continuing with MD simulations of real electrolyte environments, we analyzed the kinetic properties and solvation shell layers of LiFSI/DME electrolytes in the presence of different additives. The results show that C_60_F_4_, C_60_F_6_, and 2-C_60_F_8_ all enhance the migration of Li^+^ in the electrolyte to a certain extent, with 2-C_60_F_8_ having the most significant enhancement effect, but the introduction of other additives leads to a decrease in Li^+^ migration. Therefore, whether different fluorinated fullerenes have the prospect of practical electrolyte additive applications has to be analyzed specifically for different F atom numbers and F atom introduction sites. In conclusion, the study shows to some extent that fullerenes and their fluorinated derivatives have promising applications in the field of electrolytes. This study not only expands the application scope of fullerenes and their derivatives, but also promotes the further development of electrochemical energy storage to a certain extent.

## Figures and Tables

**Table 1:** The calculated bond length (*L*), density of all electrons (*ρ*_BCP_), energy density (*H*_BCP_), potential energy density to Lagrangian kinetic energy (|*V*_BCP_|/*G*_BCP_), and Mayer bond order (MBO) values for C-C and C-F bonding. Also shown is the NPA charge of the F atom in fullerenes and fluorinated fullerenes.

Features		C_60_	C_60_F_2_	C_60_F_4_	C_60_F_6_	1-C_60_F_8_	2-C_60_F_8_
C-C bonding	*L* (Å)	1.45	1.62	1.60	1.62	1.58	1.58
	*ρ*_BCP_	0.28	0.22	0.26	0.22	0.23	0.21
	*H*_BCP_	−0.25	−0.16	−0.21	−0.16	−0.18	−0.15
	|*V*_BCP_|/*G*_BCP_	4.11	4.67	4.60	4.67	4.87	4.71
	MBO	1.16	0.87	0.89	0.89	0.89	0.88
C-F bonding	*L* (Å)	/	1.39	1.38	1.39	1.36	1.38
	*ρ*_BCP_	/	0.24	0.24	0.24	0.25	0.24
	*H*_BCP_	/	−0.32	−0.34	−0.33	−0.35	−0.33
	|*V*_BCP_|/*G*_BCP_	/	2.12	2.05	2.07	2.04	2.07
	MBO	/	0.83	0.81	0.82	0.84	0.83
F atom	NPA charge	/	−0.37	−0.36	−0.35	−0.35	−0.35

**Table 2:** Coordination number of Li^+^ with anions (CN-Li^+^-O_FSI_^−^) and solvents (CN-Li^+^-O_DME_) in different electrolyte systems.

Electrolytes	CN-Li^+^-O_FSI_^−^	CN-Li^+^-O_DME_
LiFSI/DME	1.90	4.07
LiFSI/DME/C_60_	1.74	4.25
LiFSI/DME/C_60_F_2_	1.73	4.25
LiFSI/DME/C_60_F_4_	1.68	4.29
LiFSI/DME/C_60_F_6_	1.61	4.37
LiFSI/DME/1-C_60_F_8_	1.33	4.65
LiFSI/DME/2-C_60_F_8_	1.73	4.24

**Table 3:** Electrolyte properties simulated by molecular dynamics.

Electrolytes	Box Size (nm)	Molar Ratio	Viscosity (10^3^ μPa·s)	Diffusion Coefficient (1 × 10^7^ cm^2^/s)
LiFSI/DME	4.81 × 4.81 × 4.81	100:602	3.64	5.30
LiFSI/DME/C_60_	4.82 × 4.82 × 4.82	100:602:1	3.59	5.16
LiFSI/DME/C_60_F_2_	4.81 × 4.81 × 4.81	100:602:1	4.46	4.78
LiFSI/DME/C_60_F_4_	4.81 × 4.81 × 4.81	100:602:1	4.41	6.43
LiFSI/DME/C_60_F_6_	4.81 × 4.81 × 4.81	100:602:1	3.57	6.79
LiFSI/DME/1-C_60_F_8_	4.81 × 4.81 × 4.81	100:602:1	4.92	5.26
LiFSI/DME/2-C_60_F_8_	4.82 × 4.82 × 4.82	100:602:1	3.99	8.15

## Data Availability

The data presented in this study are available on request from the corresponding authors.
